# Milestones and Impact Factors

**DOI:** 10.1186/1476-069X-9-35

**Published:** 2010-07-08

**Authors:** David M Ozonoff, Philippe Grandjean

**Affiliations:** 1 Department of Environmental Health, Boston University School of Public Health, Boston, MA, USA; 2 Department of Environmental Medicine, University of Southern Denmark, 5000 Odense, Denmark; 3 Department of Environmental Health, Harvard School of Public Health, Boston, MA 02215, USA

## Abstract

Environmental Health has just received its first Impact Factor by Thomson ISI. At a level of 2.48, this achievement is quite satisfactory and places Environmental Health in the top 25% of environmental science journals. When the journal was launched in 2002, it was still unclear whether the Open Access publishing model could be made into a viable commercial enterprise within the biomedical field. During the past eight years, Open Access journals have become widely available, although still covering only about 15% of journal titles. Major funding agencies and institutions, including prominent US universities, now require that researchers publish in Open Access journals. Because of the profound role of scientific journals for the sharing of results and communication between researchers, the advent of Open Access may be of as much significance as the transition from handwriting to printing via moveable type. As *Environmental Health *is an electronic Open Access journal, the numbers of downloads at the journal website can be retrieved. The top-20 list of articles most frequently accessed shows that all of them have been downloaded over 10,000 times. Back in 2002, the first article published was accessed only 49 times during the following month. A year later, the server had over 1,000 downloads per month, and now the total number of monthly downloads approaches 50,000. These statistics complement the Impact Factor and confirm the viability of Open Access in our field of research. The advent of digital media and its decentralized mode of distribution - the internet - have dramatically changed the control and financing of scientific information dissemination, while facilitating peer review, accelerating editorial handling, and supporting much needed transparency. Both the meaning and means of "having an impact" are therefore changing, as will the degree and way in which scientific journals remain "factors" in that impact.

## The Impact Factor

We have just passed another milestone in the history of *Environmental Health*, the bestowal of an official Impact Factor (IF) by Thomson ISI. The idea of a quantitative measure of a journal's "impact" was suggested in 1955 by Eugene Garfield in *Science *[1,2]. Garfield's original objective was to use it as a guide for selecting journals to be included in a new reference source, what later became the Science Citation Index which was launched in 1961. This explains both the origin and some of the limitations of the IF, which subsequently morphed into a perceived measure of a scientific journal's importance to science. It is calculated for a specific year as the number of times during that year that articles from the two previous years were cited divided by the total number of citable articles in these two years. The definition of citable articles does not include Editorials such as this, although if there were any citations in this Editorial to articles published in *Environmental Health *in 2008 and 2009 they would count for this journal's 2010 Impact Factor, thus slightly inflating it.

This is not the only quirk in the calculation of IFs [3], leading some critics to question its validity [4] (to which Thomson ISI has replied [5]). Recently, there has been official recognition of the limitations of the IF for evaluating scientists for promotion or funding [6-9]. As a result, it is likely the practical significance of having an IF is less now than previously. The fact remains, however, that having an official IF is a sign that a journal has reached a mature form.

With an IF of 2.48, *Environmental Health *ranks in the top 25% of journals (44 out of 180) listed in the 'Environmental Sciences' category. Of additional importance, an impact factor above 2 is important in some countries, where the productivity of researchers is rated annually from their publications in journals with an IF above that level. We made this mark on our first attempt.

The IF we received is also more than respectable for a journal, such as ours, because the time gap between publication and citation is longer in highly specialized disciplines. Thus, the excellent performance by this metric testifies to the quality of the articles and the value of increased accessibility and timeliness afforded by open access and online publication.

A milestone is a marker along a road that shows the distance one has come, and, if the destination is known, the distance yet to be traveled. Science is open ended so there is no ultimate destination, yet we can still make some observations about the landscape we are traveling through in this transitional period in scientific publishing. But we first take a brief look back as we approach the end of our first decade as an established journal.

## The Origins of Open Access

*Environmental Health *was launched in 2002, among the very first of the independent specialty journals on the roster of a new Open Access (OA) publisher, BioMed Central (BMC), which began in 2000 with its own in-house BMC journals. Its philosophical roots can be traced further back in time, but as a practical matter it was the almost free and limitless publishing and distribution technology of the internet in the 1990 s that made the modern OA movement possible. Suddenly, the major expense of publishing a scientific article online was in actually doing the science and the time and effort of authoring. All peer review and most executive editing was also done without cost to the publisher. The value added of printing and distribution provided by conventional print publishers suddenly disappeared, but in 2002 it was not yet demonstrated that the OA publishing model could be made into a viable commercial enterprise.

The realization that the printed journals from the big publishers were no longer an essential ingredient didn't happen overnight. The technology developed much more quickly than established scholars and scientists were able to accommodate, and recognition of the new conditions was unevenly distributed across disciplines. Except for a few isolated examples in the humanities and the social sciences, it was in physics and computer science that the first change occurred. In these fields a submitted paper could take 1 to 2 years to see print and when the papers finally appeared, their complex notation was usually produced by a standard open source and free typesetting program developed by Donald Knuth called TeX (now mainly seen in the form of one of its progeny, LaTeX). Because LaTeX was widely available for desktop computers and could be obtained without cost, physicists and computer scientists could produce and share their own "camera ready" manuscripts, circulate them for comment and establish priority before publication, even before peer review. Moreover the TeX typesetting language was not only standard but used only plain text that was "marked up" with tags, much like HTML, so collaboration and sharing of work could be done easily by email. From there it was a short step to depositing the manuscript drafts essentially in publishable form in a central source, often called a preprint server. It was just such a server for physics papers that was established at Los Alamos National Laboratory in the early 1990 s. The arXiv.org server still exists and self-archiving among physicists has not only become the norm, but in some subfields like high energy-physics, it is said to be 100%. From physics the pre-print culture spread to related fields like mathematics. Computer scientists had already been doing this and today their preprint server is almost twice as big as arXiv.org, automatically harvesting preprints (and now post-print or already peer reviewed and published articles) from specialized websites and institutional repositories, Google-style.

By comparison, open and free access to electronic versions of scientific papers has come late to biomedicine and still faces opposition from large publishers and a lack of understanding from many biomedical scientists. Paradoxically, biomedicine was an early adopter of digital referencing and searching. As long ago as 1879, when John Shaw Billings made a catalog of the US Surgeon General's Library which he had been developing as a repository of medical literature, the US Government had been supporting a monthly publication listing the medical periodical literature called Index Medicus. In 1964 the Library, now called the National Library of Medicine, produced the first computerized version of Index Medicus, the Medical Literature Analysis and Retrieval System (MEDLARS), rechristened Medline in 1971 when it went "online" (at the time this meant remote access from other authorized computer systems). Medline was accessible to libraries but not to the general public until 1997, when it was made freely available in a web-based version called PubMed [10]. Suddenly the world's biomedical literature was at the fingertips of anyone with a computer connection, not just scientists with access to a medical library.

But "fingertips" didn't mean "in hand". Medline/PubMed addressed finding what was happening in the exploding world of biomedicine in the form of journal citations, but did not provide the articles themselves. That still required either a personal subscription or access to a medical library. The rapid development of biomedicine also produced a proliferation of scientific journals, many catering to highly specialized branches of basic biology or clinical subspecialty. In 1999, the NIH Director Harold Varmus suggested a combined pre-print/post-print server, but it did not find immediate acceptance, although a beginning was made in early 2000 with the establishment of a post-print (already published) archive called PubMed Central. Starting with articles from only two journals, PNAS: Proceedings of the National Academy of Sciences, and Molecular Biology of the Cell, it now includes the full text of articles from thousands of journals, including this one (for a full current list, see [11]). All BMC published articles are also archived in other national repositories (INIST, France; Koninklijke Bibliotheek, The Netherlands; PubMed Central Canada; UK PubMed Central), where they will be available whatever the fate of this or any other journal.

The importance of OA in our field is illustrated by some recent statistics collected by a project supported by the European Commission [12]. The researchers generated a bibliometric analysis of environmental health research in Europe during 1995-2005. Through the PubMed database, a total of 6,329 articles were identified. One key finding, which the authors refrained from mentioning in the abstract, was that the articles had been published in a total of 711 scientific journals [12]. Thus to follow all the research in our field, one would have to access a very large number of scientific journals, not to mention information published by other means. The internet has immensely facilitated literature reviews, but only about 15% of scientific journals are likely to be OA. In that case, a rough estimate would suggest that about 600 of the 711 journals are not OA.

## The Open Access Movement

In the early years of the 21^st ^century's first decade, the biomedical science community was still hesitant about whether OA publishers would be viewed as "second tier" venues and there was a faint suggestion that paying a processing charge automatically made it into a form of vanity publishing. But the technology and OA publishing movement, which had social and ideological roots, came together with the changing economics of commercial publishing to provide a powerful ally: librarians. This common interest was produced by what has come to be known as "the serials crisis," a chronic escalation of institutional subscription fees far in excess of inflation. A research article is unique and cannot be exchanged for a less expensive version in another journal, making price competition ineffective. The publishing industry was undergoing consolidation, so that by the early years of the millennium, a few publishers were producing a large proportion of all scholarly output.

Libraries were bearing the brunt of this cost escalation, as serials are their major expense. Any failure of budgets to keep up with rising subscription prices resulted in dropping journal subscriptions. The parent universities were also unhappy, complaining that they were paying twice for the research conducted under their auspices, once for the salaries and resources of their scientists and then again to buy back what they produced. Librarians, with either the full or tacit support of their university administrations, began to push back through active promotion of free access [13].

These economic incentives reinforced a social movement among scientists and other scholars, loosely called the Open Access movement. In 2001, 34,000 international scientists signed an Open Letter to Scientific Publishers calling for "the establishment of an online public library that would provide the full contents of the published record of research and scholarly discourse in medicine and the life sciences in a freely accessible, fully searchable, interlinked form" (text of letter:[14]; see also Open Access publishing at [15]). The arguments for Open Access publishing were many:

• most research is government funded; this implies that the public has some right of access to published research papers, its products;

• papers published as "open access" have a greater readership, on average, than those without free access [16];

• open access is almost always via electronic distribution, which is quicker and more timely than print publication and more accessible than "advanced online publication" from subscription-only sites; researchers and the public benefit from faster dissemination of research findings;

• the cost of serials is putting access to research out of reach, even for scientists, as libraries drop subscriptions; moreover not even the largest library subscribes to all journals, so specialized needs will not be serviced by traditional subscription-based publication;

• open access opens up research to new audiences, like patients with particular diseases, students and amateur scientists, and scientists in different disciplines who would otherwise not be aware of or be able to look at research in another discipline easily;

• scientists in the developing world may benefit uniquely, as many countries have no research libraries at all, let alone libraries with extensive serial holdings; to the extent that modern societies are also information-based, this reduces an important inequity

• democratic societies depend upon the participation and deliberation of its citizens which in turn depend upon their knowledge and expertise; open access and free exchange of information promotes the ability to participate and deliberate in societal decisions

*Environmental Health's *publisher, BioMed Central (BMC), began operations in 2000 as one of the first for-profit open access publishers. It now publishes over 200 journals, including this one. Most, but not all, are 100% open access journals where the author retains copyright but a form of public licensing allows free and permissionless access to anyone, providing there is attribution of credit. BMC demonstrated the commercial viability of a publishing model for peer reviewed science where the cost burden was shifted from those seeking access to those producing the science. In essence, the processing charge now covered the much smaller cost of producing the article electronically, much like the "page charge" commonly incurred in publishing in many subscription journals. Whether processing or page charges, publication charges have became just another cost of doing and disseminating research, like making slides or maintaining a service contract for instruments. It is now being routinely included (as a minor item) in grant budgets and represents a much smaller amount than standard subscription costs. A concrete symbol of the established viability of the business model came in 2008 when Springer, the world's second largest commercial publisher of science, technical and medical literature, bought BMC. BMC/Springer is continuing a policy of waiving the processing charge in cases of financial hardship or for scientists in developing countries; this decision is completely divorced from the editorial decision to publish based on peer review for scientific interest and soundness and journal scope.

With a range of OA journals available, academic institutions began to support OA publishing, and by June, 2010, 87 institutions, including prominent US universities, now require that researchers publish in OA journals. Funding institutions have a similar interest in having results from the projects they sponsor freely available to the public. Internationally, a total of 44 funding agencies require that results of their sponsored research be published in OA journals. US legislation currently allows a grace period of 12 months for the journals to charge for access, but a proposal to shorten this period has recently been put forward in the US Congress.

## Download Impact

This rapid historical review brings us to the present and the current milestone: the Impact Factor. The arcane bookkeeping and algorithmic details aside, it is surely a goal of this and every other scientific journal for its research papers to have an impact on science. Frequently papers published in *Environmental Health *are mentioned in the news (and BMC has an efficient and effective media operation to issue press releases, when appropriate), but even when our papers make news it is not *science *news, because in an important sense there is no such thing as science *news*.

Research results have an impact only by taking their place in a vast body of mutually consistent and reinforcing results. As scientists we produce pieces of a jigsaw puzzle that must be assembled into a coherent picture. Often we don't know where a piece fits, mistake a peripheral piece of the puzzle for a central one, wrongly use the fragment of a pattern as a clue to the whole, miss the meaning of a pattern because we don't see its relation to other pieces, or mistake a piece of one picture for that of another. When we are lucky or inspired we identify adjacent pieces and make them fit together or if we are exceptionally lucky we find a piece that connects whole regions of the puzzle, thus allowing us to see some of the bigger picture. The main point, however, is that it takes time for results to have an impact, the time necessary for them to be carefully put in relation to other results. Scientific research can't be "news" any more than a puzzle piece is news. It must take its place among the other puzzle pieces and be used and appreciated by the other puzzle players. For a result to be science, it has not just to be understood by a scientist -- understanding is something an individual does -- but it has to be intersubjective, something that can be shared. Publication in scientific journals is the principal means by which this sharing takes place. At least it does at this moment in time.

We have added this last qualification because this seems to be a particularly labile and uncertain period in the history of sharing and communication between people, indeed a period of profound transition, likely to be of as much historical significance as the transition from handwriting to printing via moveable type. Even before the internet, in 1962, Marshall McLuhan coined the phrase "global village" for the effect of the uniform perspective produced by printing, a format which produced multiple and identical copies of the same text that imposed the fixed view of an "author" upon a local ("village") mindset [15]. While one does not have to agree with McLuhan that this new printing technology reinforced and even produced nationalism, the dominance of rationalism and the standardization of culture and alienation of individuals, we can still see that the forced and repeatable linear arrangement of words on a printed page is a particular presentation of knowledge that affects how we learn, synthesize and represent information. The new, non-linear and inter- and cross-connected ("hyperlinked") mode of presentation of the new digital media is already changing how we see and learn things. The consequences are not predictable, but the fact that there are likely to be consequences, is.

An electronic OA journal, such as *Environmental Health*, has an added advantage: We can follow the numbers of downloads occurring at our website. As articles can also be downloaded from the PubMed Central server at the US National Library of Medicine, we count only the approximately 50% that occur directly from our own website. This information is available with the top-20 list of most frequently accessed articles at our website, and authors can track the numbers of downloads their own articles have received. The all-time top-20 list shows highly popular articles, all of them downloaded over 10,000 times from our journal server.

**Figure 1:**
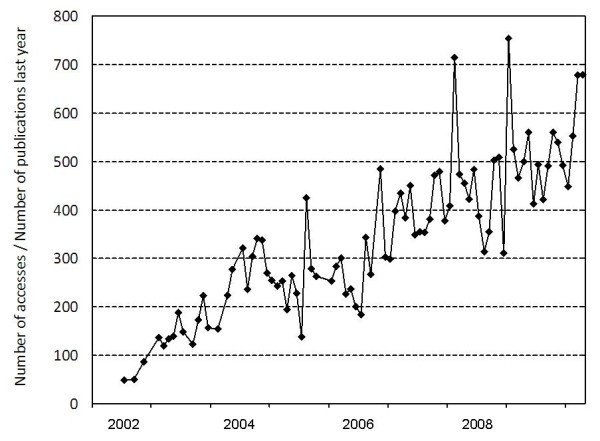
**Number of downloads from the Environmental Health journal server per month divided by the number of articles published during the previous 12 months**.

From a modest start in 2002, the first article published was only accessed 49 times during the following month. Less than a year later, we had over 1,000 downloads per month, and the total number of downloads in May, 2010 was 48,988. However, there are now 289 published articles available OA. As most downloads usually occur within the first year after publication, we therefore generated a graph that shows the total number of downloads month by month divided by the total number of articles published during the previous year (Fig. 1). From a meager score of 49 during the very first month, when only one article was available, numbers have by now increased by a factor of 10. Our recent experience suggests that a new article is usually downloaded at least 500 times per month during the first months after publication, some even more frequently. Although articles older than one year continue to be accessed, these numbers reflect the intense traffic that our web site is receiving. We believe that such statistics complement the IF, confirming the impact our journal is making.

## Further Advantages of Open Access

As we already discussed in a previous editorial [17], we take advantage of the internet facilities by using an open peer-review system and by making the reviews of accepted articles publicly available. This way, we believe that we provide a small, but well deserved recognition of the hard work contributed by our reviewers. We also believe that the added transparency makes it easier to identify possible conflicts of interest, which appear to occur much more widely than previously acknowledged [18]. According to the Institute of Medicine, conflicts constitute "a set of circumstances that creates a risk that professional judgment or actions regarding a primary interest will be unduly influenced by a secondary interest." [19]. As editors, we may not necessarily identify competing interests that should influence our own judgment of a manuscript, but by making information on competing interests and revealing the identity of reviewers, and their declarations, we believe that we facilitate this judgment.

The immediate practical consequences of OA also relate to the sudden dislocation of the locus of control for publishing and distribution and the business models for information production, dissemination and consumption. The "power of the press" not only referred to the power of the ideas conveyed by the printed word but the power of the owner of the press itself. With the advent of digital media and its decentralized mode of distribution (the internet), both the control and financing of scientific and other information dissemination has shifted dramatically, leaving conventional print publishers of periodicals and newspapers floundering as they try to adapt to a new and difficult economic landscape.

In this new land it is likely that both the meaning and means of "having an impact" will change and the degree and way in which scientific journals will be "factors" in that impact will change with them, perhaps in dramatic and unforeseen ways. While we have reached one kind of milestone in the life of what is now a mature and established scientific periodical that uses an emerging and increasingly dominant form, the journey is only begun. No one knows what the road ahead looks like, where it leads and what will be considered "milestones" as we move forward from here.

So far, it's been an adventure.

## Abbreviations

BMC: BioMed Central; IF: Impact Factor; ISI: Institute for Scientific Information; MEDLARS: Medical Literature Analysis and Retrieval System; OA: Open Access.

## Competing interests

DO and PG are founding editors-in-chief of Environmental Health.

## Authors' contributions

DO drafted the first version of the manuscript, and both authors contributed to and approved the final version.
